# Massive Open Online Course Evaluation Methods: Systematic Review

**DOI:** 10.2196/13851

**Published:** 2020-04-27

**Authors:** Abrar Alturkistani, Ching Lam, Kimberley Foley, Terese Stenfors, Elizabeth R Blum, Michelle Helena Van Velthoven, Edward Meinert

**Affiliations:** 1 Global Digital Health Unit Imperial College London London United Kingdom; 2 Digitally Enabled PrevenTative Health Research Group Department of Paediatrics University of Oxford Oxford United Kingdom; 3 Department of Learning, Informatics, Management and Ethics Karolinska Institutet Stockholm Sweden

**Keywords:** online learning, learning, computer-assisted instruction

## Abstract

**Background:**

Massive open online courses (MOOCs) have the potential to make a broader educational impact because many learners undertake these courses. Despite their reach, there is a lack of knowledge about which methods are used for evaluating these courses.

**Objective:**

The aim of this review was to identify current MOOC evaluation methods to inform future study designs.

**Methods:**

We systematically searched the following databases for studies published from January 2008 to October 2018: (1) Scopus, (2) Education Resources Information Center, (3) IEEE (Institute of Electrical and Electronic Engineers) Xplore, (4) PubMed, (5) Web of Science, (6) British Education Index, and (7) Google Scholar search engine. Two reviewers independently screened the abstracts and titles of the studies. Published studies in the English language that evaluated MOOCs were included. The study design of the evaluations, the underlying motivation for the evaluation studies, data collection, and data analysis methods were quantitatively and qualitatively analyzed. The quality of the included studies was appraised using the Cochrane Collaboration Risk of Bias Tool for randomized controlled trials (RCTs) and the National Institutes of Health—National Heart, Lung, and Blood Institute quality assessment tool for cohort observational studies and for before-after (pre-post) studies with no control group.

**Results:**

The initial search resulted in 3275 studies, and 33 eligible studies were included in this review. In total, 16 studies used a quantitative study design, 11 used a qualitative design, and 6 used a mixed methods study design. In all, 16 studies evaluated learner characteristics and behavior, and 20 studies evaluated learning outcomes and experiences. A total of 12 studies used 1 data source, 11 used 2 data sources, 7 used 3 data sources, 4 used 2 data sources, and 1 used 5 data sources. Overall, 3 studies used more than 3 data sources in their evaluation. In terms of the data analysis methods, quantitative methods were most prominent with descriptive and inferential statistics, which were the top 2 preferred methods. In all, 26 studies with a cross-sectional design had a low-quality assessment, whereas RCTs and quasi-experimental studies received a high-quality assessment.

**Conclusions:**

The MOOC evaluation data collection and data analysis methods should be determined carefully on the basis of the aim of the evaluation. The MOOC evaluations are subject to bias, which could be reduced using pre-MOOC measures for comparison or by controlling for confounding variables. Future MOOC evaluations should consider using more diverse data sources and data analysis methods.

**International Registered Report Identifier (IRRID):**

RR2-10.2196/12087

## Introduction

Massive open online courses (MOOCs) are free Web-based open courses available to anyone everywhere and have the potential to revolutionize education by increasing the accessibility and reach of education to large numbers of people [1]. However, questions remain regarding the quality of education provided through MOOCs [1]. One way to ensure the quality of MOOCs is through the evaluation of the course in a systematic way with the goal of *improvement over time* [2]. Although research about MOOCs has increased in recent years, there is limited research on the evaluation of MOOCs [3]. In addition, there is a need for effective evaluation methods for appraising the effectiveness and success of the courses.

Evaluation of courses to assess the success and effectiveness and to advise on course improvements is a long-studied approach in the field of education [4-6]. However, owing to the differences between teaching in MOOCs and traditional, face-to-face classrooms, it is not possible to adapt the same traditional evaluation methods [7,8]. For example, MOOCs generally have no restrictions on entrance, withdrawal, or the submission of assignments and assessments [7]. The methods used in Web-based education or e-learning are not always applicable to MOOCs because Web-based or e-learning courses are often provided as a part of university or higher education curricula, which are different from MOOCs per student expectations [8]. It is not suitable to directly compare MOOCs with higher education courses by using traditional evaluation standards and criteria [8].

Despite the limitations in MOOC evaluation methods, several reviews have been conducted on MOOC-related research methods, without specifically focusing on MOOC evaluations. Two recent systematic reviews were published synthesizing MOOC research methods and topics [9,10]. Zhu et al [9] and Bozkurt et al [11] recommended further research on the methodological approaches for MOOC evaluation. This research found little focus on the quality of the techniques and methodologies used [11]. In addition, a large number of studies on MOOCs examine general pedagogical aspects of the course without evaluating the course itself. Although the general evaluation of MOOC education and pedagogy is useful, it is essential that courses are also evaluated [12].

To address the gaps in MOOC evaluation methods in the literature, this systematic review aimed to identify and analyze current MOOC evaluation methods. The objective of this review was to inform future MOOC evaluation methodology.

## Methods

This review explored the following research question: *What methods have been used to evaluate MOOCs?* [13]. This systematic review was conducted according to the Cochrane guidelines [14] and reported according to the Preferred Reporting Items for Systematic Reviews and Meta-Analyses guidelines (Multimedia Appendix 1) [15]. As the review only used publicly available information, an ethics review board approval was not required. The review was executed in accordance with the protocol published by Foley et al [13].

### Eligibility Criteria

Eligible studies focused on the evaluation of MOOCs with reference to the course design, materials, or topics. The evaluation used the following population, intervention, comparator, outcome (PICO) framework for inclusion in the study:

- Population: learners in any geographic area who have participated in MOOCs [13].
- Intervention: MOOC evaluation methods. This is intended to be broad to include qualitative, quantitative, and mixed methods [13].
- Comparator: studies did not need to include a comparator for inclusion in this systematic review [13].
- Outcome: learner-focused outcomes such as attitudes, cognitive changes, learner satisfaction, etc, will be assessed [13].

Further to the abovementioned PICO framework, we used the following inclusion and exclusion criteria.

#### Inclusion Criteria

- Studies with a primary focus on MOOC evaluation and studies that have applied or reviewed MOOC evaluation methods (quantitative, qualitative, or mixed methods) [13].
- Studies published from 2008 to 2018 [13].
- All types of MOOCs, for example, extended MOOCs, connectivist MOOCs, language MOOCs, or hybrid MOOCs.

#### Exclusion Criteria

- Studies not in the English language [13].
- Studies that primarily focused on e-learning or blended learning instead of MOOCs [13].
- Studies that focused only on understanding MOOC learners such as their behaviors or motivation to join MOOCs, without referring to the MOOC.
- Studies that focused on machine learning or predictive models to predict learner behavior.

### Search Strategy

We searched the following databases for potentially relevant literature from January 2008 to October 2018: (1) Scopus, (2) Education Resources Information Center, (3) IEEE (Institute of Electrical and Electronic Engineers) Xplore, (4) Medical Literature Analysis and Retrieval System Online/PubMed, (5) Web of Science, (6) British Education Index, and (7) Google Scholar search engine. The first search was performed in Scopus. The search words and terms for Scopus were as follows: (mooc* OR “massive open online course” OR coursera OR edx OR odl OR udacity OR futurelearn AND evaluat* OR measur* OR compar* OR analys* OR report* OR assess* AND knowledge OR “applicable knowledge” OR retent* OR impact OR quality OR improv* OR environment OR effect “learning outcome” OR learning). The asterisks after the search terms allow all terms beginning with the same root word to be included in the search. The search terms were then adjusted for each database. The complete search strategy for each database can be found in the protocol by Foley et al [13] and in Multimedia Appendix 2. In addition, we scanned the reference lists of included studies.

### Selection of Studies

Two reviewers (AA and CL) independently screened the titles and abstracts of the articles for eligibility. Selected studies were identified for full-text reading. Disagreements between the reviewers were resolved by discussions with a third reviewer (EM). Few studies (<10) were discussed with a third reviewer.

### Data Extraction

The following information was extracted from each included study using a data abstraction form (Multimedia Appendix 2): (1) article title, country of the first author, and year of publication; (2) study aims; (3) evaluation: evaluation method, study design, evaluation type (evaluation of a single MOOC, multiple MOOCs, or review of a method), data collection methods, data analysis methods, and number of participants; and (4) outcome measures of the study: learner-focused outcomes and other outcomes. The studies were classified as quantitative, mixed methods, or qualitative based on the methods used. Studies were considered as mixed methods if they used a combination of qualitative or quantitative *techniques, methods, approaches, concepts, or language* in the same study [16].

### Assessment of Methodological Quality

The Cochrane Collaboration Risk of Bias Tool for randomized controlled trials (RCTs) [17] and the National Institutes of Health—National Heart, Lung, and Blood Institute quality assessment tool for cohort observational studies and for before-after (pre-post) studies with no control group [18] were used to assess the methodological quality of the included studies depending on their study design.

### Data Synthesis

We summarized the data graphically and descriptively. The evaluation results were reported according to the design thinking approach for evaluations that follows the subsequent order: (1) problem framing, (2) data collection, (3) analysis, and (4) interpretation [19].

### Problem Framing

The evaluation-focused categories in the problem framing section were determined through discussions among the primary authors to summarize study aims and objectives. The 3 categories used in the evaluation-focused categories were defined as follows:

1. The learner-focused evaluation seeks to gain insight into the learner characteristics and behavior, including metrics such as completion and participation rates, satisfaction rates, their learning experiences, and outcomes.
2. Teaching-focused evaluation studies aim to analyze pedagogical practices so as to improve teaching.
3. MOOC-focused evaluation studies aim to better understand the efficacy of the learning platform to improve the overall impact of these courses.

Further to the evaluation-focused categories, the subcategories were generated by conducting a thematic analysis of the MOOC evaluation studies’ aims and objectives. The themes resulted through an iterative process where study aims were coded and then consolidated into themes by the first author. The themes were then discussed with and reviewed by the second author until an agreement was reached.

### Data Collection Analysis and Interpretation

The categories reported in the data collection sections were all representations of what the studies reported to be the data collection method. The categorization of the learner-focused parameters was done based on how the authors identified the outcomes. For example, if authors mention that the reported outcome was measuring *learners’ attitudes to evaluate overall MOOC experience*, the parameter was recorded in the *learner experience* category. Similarly, if the authors mentioned that the reported outcome was evaluating what students gained from the course, the parameter was recorded as *longer term learner outcomes*.

## Results

In this section, we have described the search results and the methodological quality assessment results. We have then described the study findings using the following categories for MOOC evaluation: research design, aim, data collection methods, data analysis methods, and analysis and interpretation.

### Search Results

**Figure 1:**
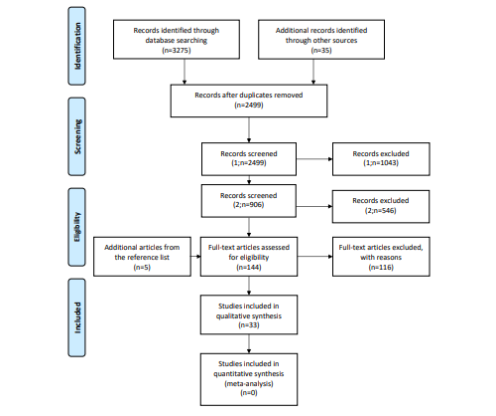
A Preferred Reporting Items for Systematic Reviews and Meta-Analyses flowchart of the literature search.

There were 3275 records identified in the literature search and 2499 records remained after duplicates were removed. Records were screened twice before full-text reading. In the first screening (n=2499), all articles that did not focus on MOOCs specifically were removed (Figure 1). In the second screening (n=906), all articles that did not focus on MOOC learners or MOOC evaluation methods were removed (Figure 1). This was followed by full-text reading of 154 studies (Figure 1). An additional 5 studies were identified by searching the bibliographies of the included studies. In total, 33 publications were included in this review. There were 31 cross-sectional studies, 1 randomized trial, and 1 quasi-experimental study. The completed data abstraction forms of the included studies are in Multimedia Appendix 3.

### Methodological Quality

The RCT included in this study [20] received a low risk-of-bias classification (Multimedia Appendix 4).

Of the 31 cross-sectional studies, 26 received poor ratings because of a high risk of bias (Multimedia Appendix 5). The remaining 5 studies received a fair rating because of a higher consideration for possible bias. In total, 2 studies that were able to measure exposure before outcomes such as studies that performed pretests and posttests [21,22], 3 studies that accounted for confounding variables [21-23], 2 studies that used validated exposure [24,25], and 2 studies that used outcome measures [23,25] received a better quality rating.

A quality assessment of the quasi-experimental study using longitudinal pretests and posttests [26] is included in Multimedia Appendix 6.

### Massive Open Online Course Evaluation Research Design

In total, 16 studies used a quantitative study design, 11 studies used a qualitative study design, and 6 studies used a mixed methods study design. There was 1 RCT [20] and 1 quasi-experimental study [26]. In total, 4 studies evaluated more than 1 MOOC [27-30]. In all, 2 studies evaluated 2 runs of the same MOOC [31,32], and 1 study evaluated 3 parts of the same MOOC, run twice for consecutive years [33].

In total, 6 studies used a comparator in their methods. A study compared precourse and postcourse surveys by performing a chi-square test of changes in *confidence, attitudes, and knowledge* [34]. A study compared the average assignment and final essay scores of MOOC learners with face-to-face learners and calculated 2 independent sample *t* tests to compare the differences between learners but did not include any pre- and posttest or survey results [35]. In all, 4 studies conducted pretest and posttest analyses [20,26]. Hossain et al [20] used an RCT design and calculated the mean between-group differences of knowledge, confidence, and satisfaction comparing MOOC learners with other Web-based learners. Colvin et al [21] calculated normalized gain using item response between pretest and posttest scores and the Item Response Theory for weekly performance compared with that of on-campus learners. Rubio et al [26] compared the pretest mean and posttest mean of comprehensibility scores in a MOOC, comparing results with those of face-to-face learners [26]. Konstan et al [22] calculated knowledge test gains by performing a paired *t* test of average knowledge gains, comparing these gains with those of face-to-face learners and (comparing 2 learner groups) the average normalized learning gains among all learners [22].

### Aim of Massive Open Online Course Evaluations

The aim or objective of MOOC evaluations included in this review can be categorized into learner-focused, teaching-focused, and MOOC-focused evaluation aims (Table 1). In all, 16 studies evaluated learner characteristics and behavior and 20 studies evaluated learning outcomes and experiences. One of the least studied aspects of MOOC evaluation is pedagogical practices, which were only evaluated by 2 studies [36,37].

**Table 1:** The aim of the massive open online course evaluations for the included studies.

Evaluation aim focus, subcategories		Studies	Number of studies
**Learner**			
	Learner expectations	[23,38,39]	3
	Learner characteristics and behavior	[22,23,27,28,30-32,38,39,40-46]	16
	Learner engagement	[27,34,43,45]	4
	Participation or completion rates	[24,30,34,42,44,45]	6
	Learner satisfaction	[20,47]	2
	Peer interaction	[25,43,46,48]	4
	Learning outcomes and experience	[21-26,31,33,35,37,39-41,43,44,46-50]	20
	Knowledge retention	[22]	1
**Teaching**			
	Pedagogical practices	[36,37]	2
**MOOC^a^**			
	Comparison with other learning platforms	[20,22,26,38]	4
	MOOC content and structure	[48]	1
	Implementation of MOOC	[29,31,33,49,52]	5
	Sustainability of MOOC	[49]	1

^a^MOOC: massive open online course.

### Massive Open Online Course Evaluation Data Collection Methods

In all, 12 studies used 1 data source [20,24,25,27,28,31,32,38,43,47], 11 studies used 2 data sources [21,26,29,30,33,34,36,40,42,45,51], 7 studies used 3 data sources [22,35,39,41,44,46,48], 2 studies used 4 data sources [23,37], and 1 study used 5 data sources [52]. The most used data sources were surveys followed by learning management system (LMS), quizzes, and interviews (Table 2). “Other” data sources that are referred to in Table 2 include data collected from social media posts [37], registration forms [30,44], online focus groups [37], and homework performance data [21]. These data sources were used to collect data on different aspects of the evaluation.

**Table 2:** Studies using different data sources (N=33).

Data source	Value, n (%)
Surveys	20 (30.8)
Interviews	8 (12.3)
Learning Management System	18 (27.7)
Discussions	5 (7.7)
Quizzes	9 (13.8)
Other	5 (7.7)

In total, 8 studies collected data through interviews and had a population size ranging from 2 to 44 [23,37,39,42,43,49,51,52]. In total, 20 studies that collected data through surveys had a population size ranging from 25 to 10,392 [22-41,44-46,51,52]. In all, 18 studies that collected data through the LMS [22,24,26,29-31,33-35,39-42,44-46,48,52] had a population size made of participants or data points (eg, discussion posts) ranging from 59 to 209,871. Nine studies used quiz data [20-22,26,33,35,41,47,52]. Studies that used quiz data had a population size of 48 [20], 53 [47], 136 [41], 1080 [21], and 5255 [22]. Other data sources used did not have a clearly reported sample size for a particular source.

Table 3 shows the various data collection methods and their uses. Pre-MOOC surveys or pretests could be used for baseline data such as learner expectations [22,36,50] or learner baseline test scores [20-22,26,33], which, then, allows tests scores to be compared with post-MOOC survey and quiz data [20-22,27,33]. Table 3 explains *how* studies collected data to meet the aims of their evaluation. In general, surveys were used to collect demographic data, learner experience, and learner perceptions and reactions, whereas LMS data were used for tracking learner completion of the MOOCs.

**Table 3:** Data collection methods and their uses in massive open online course evaluations.

Data	Uses
Registration form	To collect demographic information [26,30]
Pre-MOOC^a^ survey	To collect data on the following: demographic information [23,29,36,40,46,50,52]; learners’ background [22,29,36,46] and expectations; perceptions [22,36,50]; learners’ experience [40]; learners’ past MOOC experience [29]; learners’ self-efficacy [52], motivation [52], and goals [44,50]; assess learners’ knowledge [40] and course efficacy [50]
Pretest	To collect baseline test scores for comparison with posttest scores [20-22,26,33]
Learning management system data	To collect data on the following: demographic information [24]; attendance rates [24,35,42]; completion of the different components of the MOOC [24,35,36,39,42]; quiz or assignment scores [26,35,45]; learner activity [45]
Discussion posts	Feedback about the course [46] and learner interactions [25]
Quiz, homework, or test (not specified as pre- or postquiz or test)	Grades to assess learning [21,35,41] and a weekly quiz to record learners’ reaction to the tools called *digital readiness tools* of the course [47]
Post-MOOC survey	To collect demographic information [23,39-41,50,51]; to record the learning experience [22,30,35,39,41]; to record course influence [48]; to guide MOOC design [48], course feedback [30,33,39,41,46,50], perceptions [38,50], excitement [38], learners’ motivation [23,32,39], learners’ satisfaction [35,40,41], enjoyment of the course [40] and “Patterns and levels of participation” in the course [37], learning strategies [32]; to assess learner knowledge [40], course usefulness [39,45], course degree of perseverance [45], reasons for dropping out of the MOOC [30]; to recruit participants for research [23]; to collect course feedback [44]
Posttest	To assess learning [20,21]; to assess confidence in applying learning [20]; to assess satisfaction [20]; to calculate the difference in scores compared with pretest [22,26,33]; to assess knowledge retention 5 months post-MOOC [22]
End of MOOC quiz	To record learners’ feedback in relation to the course material (whether the course helped them become *flexible learners*) [47]
Postcourse interview	Course participation and evaluation [37,42]; course effectiveness [43,49]; sustainability of the course [49]; reason for taking the course [23]; learners’ motivation [23,42]; to understand learning behavior [51]; *postcourse practices* or learners’ behavior [52]
Email interview	To understand learners’ behavior and learning in MOOCs [37]; specify MOOC positives [39]; motivation in MOOC; challenges in MOOC [39]
Online focus group	Assessment of the course: organization, assessment, *use of technology* and *inclusive practice* [37]

^a^MOOC: massive open online course.

### Massive Open Online Course Evaluation Analysis and Interpretation

In terms of the data analysis methods, quantitative methods were the only type of method used in 16 studies with descriptive and inferential statistics, the top 2 preferred methods. Qualitative analysis methods such as thematic analyses, which can include grounded theory [49], focused coding [38,39], and content analysis [25,50], were mainly used in qualitative studies.

A summary of the parameters, indicators, and data analysis used for the MOOC evaluation can be found in Table 4. Most notably, inferential statistics were used to analyze learning outcomes (Table 4) such as the comparison of means or the use of regression methods to analyze quiz or test grades. These outcomes were also used as a measure to evaluate the overall effectiveness of a MOOC by the studies. Table 4 shows how the data collection method uses mentioned in Table 3 were measured and analyzed. In general, studies focused on measuring learner engagement and learners’ behavior–related indicators. Studies referred to learning in different ways such as *learning*, learning performance, learning outcome, or gain in comprehensibility depending on the learning material of the course. Other studies considered learning outcomes such as knowledge retention or *what students took away from the course*. There was a consensus that learner engagement can be measured by measuring the various learner activities in the course, whereas learner behavior was a more general term used by studies to describe the different MOOC evaluation measures. For teaching-focused evaluation, both Mackness et al [37] and Singh et al [36] used learner parameters to reflect and analyze pedagogical practices.

**Table 4:** Data collection method uses mentioned earlier and how they were analyzed in massive open online course evaluations.

Data collection method uses, parameters or themes reported		Data analysis methods
**To measure learning outcomes**		
	Learning [20,33] Learning performance [52] “Learning outcome” [41] Learning [21] Overall learner ability in the course [21] The students’ gains in comprehensibility [26] Subject-matter knowledge [22] Comprehensibility of learner audio recordings in a language MOOC^a^ [26] Knowledge retention [22] Learning performance [35]	Calculation of the mean difference between pretest and posttest scores [20,33] Compare pretest and posttest scores using a paired *t* test [33] Descriptive statistics [52] “Regressing quiz and homework score on participation and MOOC experience” [41] Calculation of normalized gain between pretest and posttest Using the Item Response Theory analyzing pretest, posttest, and homework performance [21]A matched-pairs *t* test to measure the “gains in comprehensibility between the pretest and posttest” and an unpaired *t* test to compare the pretest and posttest means [26] Knowledge test gains by calculating normalized learning gains when comparing pretest and posttest scores [22] Calculation of gains in comprehensibility [26] “A paired-samples *t* test to examine student knowledge retention as measured by the postcourse and follow-up tests” [22] Two independent sample *t* tests to compare the quiz and assignment scores [35]
**To measure learner participation or engagement**		
	Contributions per week, number of tweets, “quality of posted comments and learning designs,” “quality of peer feedback,” “ranking of importance of course features,” “comments received by those posting and sharing a scenario idea in Week 2” [35,44] Determinants of completion [22] Course completion rate [42] The number of videos watched, video activity (play, stops, and full watch), the number of quizzes submitted, and discussion forum activity; reading in forums, the number of posts and comments, and dropout rate [31] Reasons for dropping out of the course [30], total number of reads in forums, the number of forum and post comments [31] The number of comments per participant, completed steps, and the “likes” count [34] The frequency of viewing lectures [40] and frequency of attempting quizzes [40] Learner course activity and course grade [45] and frequency of interaction on online forums Satisfaction with MOOC, comfort with learning new things, and joining MOOC because of the “Love for Learning” [27]	Descriptive statistics [30,31,34,35,42,44] and frequency analysis [45] Logistic regression of homework and exam outcomes [22] Regression [40] Frequency analysis [45] and structural equation modeling [27]
**To measure learner experience**		
	Comparison of a Likert scale rating of “the technology quality and user-friendliness of the Web environment, the quality of instructional content, and the instructional arrangement,” satisfaction with interactions with instructors, satisfaction with support received, and the satisfaction of learning needs between MOOC and onsite learners [35] “Perceived usefulness and ease of use” of MOOC [41] “Perceived learning experience” [41] Learner rating of the “usefulness and relevance of the activities” [45] Overall learner attitude [23]	Comparison of “Likert scale items” using the Mann-Whitney U tests [35], regression analysis [41], factor analysis of factors related to *poststudy feedback* [41], frequency analysis [45], sentiment analysis of interview data [23], and descriptive statistics [30]
**To measure learner expectation**		
	Student expectations (theme) [50] Whether course fulfilled expectations [30]	Descriptive content analysis [50] Descriptive statistics [30]
**To measure learner behavior**		
	“Autonomous learning across distributed platforms, learning through diversity, learning through openness and interactivity, organizing learning through aggregation, co-creation, and creativity through remixing and repurposing, coping with uncertainty, and identity building” (themes) [37] How learners approach “professional learning” in a MOOC, what learner behavior is exhibited by learners, and how “professionals relate their MOOC learning to their professional role” [51] Factors predicting learner and student success [22] Learner self-reported “assertions on learning strategies” [32]	Qualitative descriptive [37] Coding of interview data [51] Ordinary least squares regression using learner demographic data and knowledge data [22] Descriptive statistics [32]
**To measure learner retention**		
	Learner course activity and course grade [45] Learner rating of course perseverance [45]	1-2 frequency analysis [45]
**To measure long-term learner outcomes**		
	Learner opinions about course effectiveness [49] “What students took away from the MOOC” (theme) [50]	Using grounded-theory methods of interview data [49] Descriptive content analysis [50]
**To measure social interactions**		
	Learner “interaction in forums” [42] Learner to learner interactions [25] Learner collaboration patterns [25]	Social network analysis [42] Content analysis of discussions posts using the Interaction Analysis Model [25] Social network analysis [25]
**To measure learner motivation**		
	“Learning motivation” [42] Learner self-reported “assertions on motivation” [32] “A reason for taking or completing the course” [23] Exploring the “primary motivation for taking” the course [28]	Descriptive qualitative [42] Descriptive statistics [32] Thematic analysis of interview data [23] Emergent coding on survey data [28]

^a^MOOC: massive open online course.

## Discussion

This study aimed to review current MOOC evaluation methods to understand the methods that have been used in published MOOC studies and subsequently to inform future designs of MOOC evaluation methods. Owing to the diversity of MOOC topics and learners, it is not possible to propose a single evaluation method for all MOOCs. Researchers aiming to evaluate a MOOC should choose a method based on the aims of their evaluation or the parameters they would like to measure. In general, data collection methods were similar in most evaluations, such as the use of interviews or survey data, and the analysis methods were highly heterogeneous among studies.

### Massive Open Online Course Evaluation Research Design

The cross-sectional study design was used in 31 of 33 of the included studies. The cross-sectional study design was used when the aim was to investigate the factors affecting outcomes for a population at a given time point [53]. For the MOOC evaluation, this is particularly useful for observing the population of learners and for understanding the factors affecting the success and impact of a MOOC. They are relatively inexpensive to conduct and can assess many outcomes and factors at the same time. However, cross-sectional study designs are subject to *nonresponse bias*, which means that studies are only representative of those who participated, who incidentally may happen to be different from the rest of the population [53].

One of the most effective methods of evaluation used in MOOCs was the use of baseline data to compare outcomes. Studies that did pretests and posttests had a less likelihood of bias in their outcomes owing to the measurement of exposure before the measurement of outcome [18]. Even when studies used pre- and postcourse surveys or tests, they were not longitudinal in design, as such a design requires a follow-up of the same individuals and requires observing them *at multiple time points* [53]. Therefore, the use of pre- and postsurveys or tests without linking the individuals may simply represent a difference in the groups studied rather than changes in learning or learner outcomes. The advantages of this method are that it can reduce bias, and quasi-experimental studies are known as strong methods. However, the disadvantage is that although this method may work with assessing learning, such as memorizing information, it may not work to assess skill development or the application of skills.

### Aim of Massive Open Online Course Evaluations

Understanding the aim behind the evaluation of MOOCs is critically important in designing MOOC evaluation methods as it influences the performance indicators and parameters to be evaluated. More importantly, motivation for the evaluation determines the data methods that will be used. One reason for the inability to conclude a standardized evaluation method from this review is that studies differ in the aspects and purposes of why they are conducting the evaluation. For example, not all studies perform evaluations of MOOCs to evaluate overall effectiveness, which is an important aspect to consider if MOOCs are to be adopted more formally in higher education [54]. The variability in the motivation of MOOC evaluations may also explain the high variability in the outcomes measured and reported.

### Data Collection Methodology

In all, 12 studies used 1 data source and 11 studies used 2 data sources (Table 3), which is not different from previous findings [10]. The results of this study also show that there is high flexibility in data collection methods for MOOC evaluations from survey data to LMS data to more distinct methods such as online focus groups [37]. The number of participants in the studies was exceedingly varied. This is due to the difference in the data collection methods used. For example, studies with data captured through the LMS, which is capable of capturing data from all of the learners who joined the course, had the highest number of learners. On the contrary, studies that used more time-consuming methods, such as surveys or interviews, generally had a lower number of participants. It is important to note that the MOOC evaluation is not necessarily improved by increasing the number of data sources but rather by conducting a meaningful analysis of the available data. Some studies preferred multiple methods of evaluation and assessment of learning. One paper argued that this allows to evaluate learning of the diverse MOOC population in a more effective way [22]. Studies should use the best data collection methods to answer their research aims and questions.

### Analysis and Interpretation

In total, 16 of 33 studies used only quantitative methods for analysis (Table 4), which is in line with the general MOOC research, which has been predominated by quantitative methods [10,55]. Studies used statistical methods such as descriptive and inferential statistics for data analysis and interpretation of results. The availability of data from sources such as the LMS may have encouraged the use of descriptive statistical methods [10]. However, 17 of the 33 included studies used some form of qualitative data analysis methods either by using a qualitative study design or by using a mixed methods study design (Table 4). This may be explained by the recent (2016-2017) rise in the use of qualitative methods in MOOC research [10].

Although inferential statistics can help create better outcomes from studies, this is not always possible. For example, one study [36] mentioned a high variation between pre- and postcourse survey participant numbers and another [29] mentioned a small sample size as reasons for not using inferential statistical methods. It should be noted that using data from multiple sources and having a large sample size does not guarantee the quality of the evaluation methods.

In MOOC research, qualitative data can be useful to understand the meaning of different behaviors as quantitative data, oftentimes, cannot answer why things happened [56].

Thematic and sentiment data analysis methods seek to represent qualitative data in a systematic way. The thematic analysis seeks to organize information into themes to find patterns [57]. This is especially useful for generalizing data for a subsequent analysis. For instance, Singh et al [36], Draffan et al [34], and Shapiro et al [23] all used a thematic analysis to simplify heterogeneous responses from interviewees and participants to understand what students enjoy about the MOOCs. Focused coding and grounded theory use similar approaches to grouping qualitative data into themes based on conceptual similarity and to developing analytic narratives. Liu et al [38] used focused coding to group data from course surveys into positive and negative aspects of MOOCs for future MOOC improvement [7]. Sentiment analysis and social network analysis are both qualitative analysis strategies with a greater focus on opinion-rich data [58]. These are important strategies used in understanding the opinions of learners and converting subjective feelings of learners into data that can be analyzed and interpreted.

### Outcome Measures

The outcome measures reported greatly varied among studies, which is expected, as identifying the right outcome measures is an inherent challenge in educational research, including more traditional classroom-based studies [7].

The choice of evaluation methods is highly dependent on the aim of the evaluation and the size of the MOOCs. For quantitative measures, such as completion and participation rates, metrics can be easily collected through the MOOC platform. However, these metrics alone may be insufficient to provide insights into why students fail to complete the course for future improvement. Although it may be difficult to represent the problem holistically using qualitative methods, it can be useful in providing insights from individuals who participated in the MOOCs. Mixed methods studies combine the 2 modalities to better understand metrics generated and produce greater insights for future improvement of the MOOCs.

Learning outcomes were mostly analyzed by inferential statistical methods owing to the use of pretest and posttest methods and the calculation of gains in learning. This method may be most suited for MOOCs that require knowledge retention. Learning parameters also involved a lot of comparisons, either a comparison with pre-MOOC measures or a comparison with other learners or both. Social interactions were studied in 2 of the MOOC evaluations using social network analysis methods. Although the MOOC completion rate has been often cited as a parameter for MOOC success, it can be noticed that studies started to move away from only using completion rates. For example, studies looked at completion of different steps of the MOOCs or looked at overall completion. The learning outcomes reported in this review should be used with caution as not all of them have been validated or assessed for their reliability except for a few.

### Methodological Quality

In total, 26 studies with a cross-sectional design had a low-quality assessment, whereas RCTs and quasi-experimental studies received a high-quality assessment. Having a high level of bias affects the generalizability of studies, which is a common problem in most research using data from MOOCs [30,59]. The availability of high risks of bias in current MOOC evaluations requires a closer look at what were the sources of bias and what methods can be used to reduce them. The use of not validated, self-reported data sources and the lack of longitudinal data also increases the risk of bias in these studies [56]. However, although most MOOCs struggle with learner retention and MOOC completion rates [54], it is understandable that studies are not able to collect longitudinal data.

### Future Directions

The scarcity of studies focusing on the evaluation of the effectiveness of particular MOOCs relative to the number of available studies on MOOCs raises some questions. For example, many studies that were excluded from this review studied MOOC learners or aspects of the MOOCs without conducting an evaluation of course success or effectiveness. As shown in this review, there is a diverse range of evaluation methods, and the quality of these evaluation studies can be as diverse. The motivation of the evaluation exercise should be the basis of the evaluation study design to design effective quantitative or qualitative data collection strategies. The development of general guidance, standardized performance indicators, and an evaluation framework using a design thinking approach can allow these MOOC evaluation exercises to yield data of better quality and precision and allow improved evaluation outcomes. To provide a comprehensive evaluation of MOOCs, studies should try to use a framework to be able to systematically review all of the aspects of the course.

In general, the adoption of a mixed methods analysis considering both quantitative and qualitative data can be more useful for evaluating the overall quality of MOOCs. Although it is useful to have quantitative data such as learner participation and dropout rates, qualitative data gathered through interviews and opinion mining provide valuable insights into the reasons behind the success or failure of a MOOC. Studies of MOOC evaluations should aim to use data collection and analysis methods that can minimize the risk of bias and provide objective results. Whenever possible, studies should use comparison methods, such as the use of pretest or posttest or a comparison with other types of learners, as a control measure. In addition, learner persistence is an important indicator for MOOC evaluation that needs to be addressed in future research.

### Strengths and Limitations

To our knowledge, this is the first study to systematically review the evaluation methods of MOOCs. The findings of this review can serve future MOOC evaluators with recommendations on their evaluation methods to facilitate better study designs and maximize the impact of these Web-based platforms. However, as a lot of MOOCs are not necessarily provided by universities and systematically evaluated and published, the scope of this review can only reflect a small part of MOOC evaluation studies.

### Conclusions

There is no one way of completing a MOOC evaluation, but there are considerations that should be taken into account in every evaluation. First, because MOOCs are very large, there is a tendency to use quantitative methods using aggregate-level data. However, aggregate-level data do not always tell why things are happening. Qualitative data could further help interpret the results by exploring why things are happening. Evaluations lacked longitudinal data and very few accounted for confounding variables owing to data collection challenges associated with MOOCs such as not having longitudinal data or not having enough data sources. Future studies could help identify how these challenges could be overcome or minimized.

LMS may not report useful findings on an individual level, but they should still be considered and used in MOOC evaluations. Big data in the form of learning analytics can help with decision making, predicting learner behavior, and providing a more *comprehensive* picture of the phenomena studied [60]. Studies should still consider using LMS as it can provide a valuable addition to the research, but researchers need to be careful about the depth of the findings that can be concluded from LMS-only datasets.

The use of qualitative data could help enhance the findings from the studies by explaining the phenomena. Both quantitative and qualitative methods could play a key role in MOOC evaluations.

Current MOOC evaluations are subject to many sources of bias owing to the nature of the courses being open and available to a very large and diverse number of participants. However, methods are available to reduce the sources of bias. Studies could use a comparator, such as pretest scores, or other types of learners to be able to calculate relative changes in learning. In addition, studies could control for confounding variables to reduce bias.

This review has provided an in-depth view of how MOOCs can be evaluated and explored the methodological approaches used. Exploring MOOC methodological approaches has been stated as an area for future research [10]. The review also provided recommendations for future MOOC evaluations and for future research in this area to help improve the quality and reliability of the studies. MOOC evaluations could contribute to the development and improvement of these courses.

## Appendix

Multimedia Appendix 1 Preferred Reporting Items for Systematic Reviews and Meta-Analyses 2009 checklist.

Multimedia Appendix 2 Search strategy.

Multimedia Appendix 3 Data abstraction form.

Multimedia Appendix 4 Quality assessment results of the Randomized Controlled Trial [20] using the Cochrane Collaboration Risk of Bias Tool.

Multimedia Appendix 5 Quality assessment results of cross-sectional studies using the NIH - National Heart, Lung and Blood Institute quality assessment tool.

Multimedia Appendix 6 Quality assessment results for the quasi experimental study using the Cochrane Collaboration Risk of Bias Tool for Before-After (Pre-Post) Studies With No Control Group.

## Abbreviations

IEEE: Institute of Electrical and Electronic Engineers
LMS: learning management system
MOOC: massive open online course
PICO: population, intervention, comparator, outcome
RCT: randomized controlled trial
