# Supplementary material for: Massive Open Online Course Evaluation Methods: Systematic Review
Source: J Med Internet Res. 2020 Apr 27;22(4):e13851. doi: 10.2196/13851 (PMC7215503; doi:10.2196/13851)
Supplement: Multimedia Appendix 2 [file jmir_v22i4e13851_app2.docx]

**Multimedia Appendix 2:**

Search strategy

This search strategy has been published as part of the review protocol [13].

**Scopus:**

( TITLE-ABS-KEY ( mooc*  OR "massive open online course"  OR coursera OR edx OR odl OR  udacity OR futurelearn ) AND TITLE-ABS-KEY ( evaluat*  OR measur* OR compar* OR analys* OR report* OR assess* )  AND TITLE-ABS-KEY ( knowledge OR "applicable knowledge" OR retent*  OR impact OR quality OR improv* OR environment OR effect "learning outcome"  OR learning ) )

Limit 2008 to present

Results: 1489

**Ovid:**

(MOOC Or massive open online course OR coursera or Udacity or futurelearn OR edx and MOOC And EVALUATION STUDIES/ OR evaluat$ or measur* or compar* or analys* or report* or assess*) And (knowledge  OR KNOWLEDGE/ OR Educational measurement/ or learning outcome/ or recent or impact or quality or improv” or environment or effect OR learn or Learning/)

Limit 2008 to present

Results: 65

Notes: The Ovid search did not include edx alone because it resulted in a large number of irrelevant studies related to cells.

**ERIC:**

(mooc*  OR "massive open online course"  OR coursera OR edx OR odl OR  udacity OR futurelearn)

Limit 2008 to present

Results: 1131

Notes: The ERIC search was kept more general than other databases because adding more terms significantly limited the search.

**Web of science:**

Indexes=SCI-EXPANDED, SSCI, A&HCI, CPCI-S, CPCI-SSH, ESCI Timespan=All years

# 1 TS=(evaluate* OR measure* OR compare* OR analys* OR report* OR assess*)

# 2 TS=(Knowledge OR “applicable knowledge” OR retention OR impact OR quality OR improve OR environment OR effect OR participation OR completion OR learning OR learn)

# 3 TI=(MOOC or "Massive open online course")

# 4 88 Ti=(coursera OR odl OR udacity OR futurelearn)

# 5 1,199 #4 OR #3

# 6 #5 AND #2 AND #1

Limit 2008 to present

Results: 479

**British Education Index**

(mooc* OR "massive open online course" OR coursera OR edx OR odl OR udacity OR futurelearn) AND (evaluate* OR measure* OR compare* OR analys* OR report* OR assess*) AND (Knowledge OR “applicable knowledge” OR retention OR impact OR quality OR improve OR environment OR effect OR participation OR completion OR learning OR learn)

Limit 2008 to present

Results: 111
