# Supplementary material for: Massive Open Online Course Evaluation Methods: Systematic Review
Source: J Med Internet Res. 2020 Apr 27;22(4):e13851. doi: 10.2196/13851 (PMC7215503; doi:10.2196/13851)
Supplement: Multimedia Appendix 3 [file jmir_v22i4e13851_app3.docx]

**Multimedia Appendix 3:**

Data abstraction form

Elements of the data abstraction form that were already presents in the results section of this review were eliminated from this form.

| Study | Publication date | Country of the first author | Evaluation method | Data collection method(s) | Main data analysis method | Secondary analysis method (if any) | Group size where data was drawn from |
| --- | --- | --- | --- | --- | --- | --- | --- |
| Li Q, Wan F. 2016 | 2016 | China | Mixed-methods | -Interview  -LMS data | Social network analysis | N/A | LMS data (n=206), Interview (n=5) |
| Hossain MS, Shofiqul Islam M, Glinsky JV, et al. 2015 | 2015 | Bangladesh | Quantitative | -Quiz or assessment grades | Inferential statistics | N/A | Quiz (n=48) |
| Lei C-U, Hou X, Kwok TTO, et al. 2015 | 2015 | China | Mixed-methods | -Pre-course survey  -Post-course or end of course survey  -Discussion post or learner comments  -LMS data | Sentiment analysis | N/A | Pre-course survey (n=NS), post-course or end of course survey (n=NS), discussion post or learner comments (n=1062), LMS data (n=NS) |
| Singh AB, Mørch AI. 2018 | 2018 | Norway | Mixed-methods | -Pre-course survey  -Post-course or end of course survey  -Discussion post or learner comments | Thematic analysis | N/A | Pre-course survey (n=936), post-course survey (n=38) |
| Liang D, Jia J, Wu X, et al. 2014 | 2014 | China | Quantitative | -Post-course or end of course survey -LMS data -Quiz or assessment grades | Inferential statistics | N/A | Post-course or end of course survey, LMS data and Quiz or assessment grades (n=136) |
| Study | Publication date | Country of the first author | Evaluation method | Data collection method(s) | Main data analysis method | Secondary analysis method (if any) | Group size where data was drawn from |
| Chen W, Jia J, Miao J, et al, 2015 | 2015 | China | Quantitative | -Post-course or end of course survey  -LMS data  -Quiz or assessment grades | Inferential statistics | N/A | Survey (n=299) |
| Lin J, Cantoni L. 2017 | 2017 | Switzerland | Qualitative | -Pre-course survey  -Post-course or end of course survey  -Interview  -LMS data  -Other (social media data) | Other qualitative | N/A | Pre-course (n=477), post-course (n=114), engagement survey (n=216), in-course analytics data (n=5519), other data (n=1817), interview (n=9) |
| Alemán de la Garza LY, Sancho-Vinuesa T, Gómez Zermeño MG. 2015 | 2015 | Mexico | Quantitative | -LMS data | Descriptive statistics | N/A | LMS (n=209,871) |
| Study | Publication date | Country of the first author | Evaluation method | Data collection method(s) | Main data analysis method | Secondary analysis method (if any) | Group size where data was drawn from |
| Khalil M, Ebner M. 2018 | 2018 | Netherlands | Quantitative | -Other survey  -LMS data  -Discussion post or learner comments  -Quiz or assessment grades | Descriptive statistics | N/A | LMS 2015 data (n=368), LMS 2016 data (n=106) |
| Draffan EA, Leon M, James A, et al. 2018 | 2018 | UK | Mixed-methods | -LMS data  -Discussion post or learner comments | Thematic analysis | N/A | LMS data (n=4185), Discussion post or learner comments (n=260 comments) |
| Kaveri A, Gunasekar S, Gupta D, et al. 2016 | 2016 | India | Quantitative | -Other survey | Inferential statistics | N/A | Survey (n=59) |
| Alturkistani A, Car J, Majeed A, et al. 2018 | 2018 | UK | Qualitative | -Interview | Thematic analysis | Other qualitative | Interview (n=2) |
| Lesjak B, Florjan V. 2014 | 2014 | Slovenia | Mixed-methods | -Pre-course survey  -Post-course or end of course survey  -Other survey | Descriptive statistics | Other qualitative | Pre-course survey (n=39), post-course or end of course survey (n=39), Other survey (n=39), LMS data (n=44) |
| Study | Publication date | Country of the first author | Evaluation method | Data collection method(s) | Main data analysis method | Secondary analysis method (if any) | Group size where data was drawn from |
| Cross, Simon 2013 | 2013 | UK | Mixed-methods | -Pre-course survey  -Post-course or end of course survey  -Other survey  -LMS data | Descriptive statistics | Other qualitative | Registration form (n=3961), Pre-course Survey of Expectations (n=66), Previous MOOC Experience Survey (n=41), Post-course Survey (n=22), Feedback forms submitted during MOOC (n=31) |
| Liu M, Kang J, McKelroy E. 2015 | 2015 | USA | Mixed-methods | -Post-course or end of course survey | Thematic analysis (Focused coding) | Descriptive statistics | Post course survey (n=320) |
| Brunton J, Brown M, Costello E, et al, 2017 | 2017 | Ireland | Quantitative | -Quiz or assessment grades | Descriptive statistics | N/A | Weekly Digital Readiness Tools quiz (n=28), End of Course Quiz (n=25) |
| Mackness J, Waite M, Roberts G, et al, 2013 | 2013 | UK | Qualitative | -post-course survey  -Interview  -LMS data -Other (social media data and online focus groups) | Other qualitative | N/A | Quisttionare (n=21), face-to-face Interviews (n=4), further survey (n=27), email interviews (n=7) |
| Study | Publication date | Country of the first author | Evaluation method | Data collection method(s) | Main data analysis method | Secondary analysis method (if any) | Group size where data was drawn from |
| Colvin KF, Champaign J, Liu A, et al, 2014 | 2014 | USA | Quantitative | -Quiz or assessment grades  -Other (Homework performance) | Inferential statistics | N/A | Pretest, posttest and homework data (n=1080) |
| MacKay JRD, Langford F, Waran N, 2016 | 2016 | UK | Mixed-methods | -Pre-course survey  -Post-course or end of course survey  -LMS data | Inferential statistics | N/A | Pre-course suurvey (n=3268), post-course survey (n=2397) |
| Stephens M, Jones KML, 2014 | 2014 | USA | Qualitative | -Pre-course survey  -Post-course or end of course survey | Thematic analysis (Content analysis) | N/A | Pre-course survey (n=196), post-course survey (n=151) |
| Warriem JM, Murthy S, Iyer S, 2016 | 2016 | India | Quantitative | -Post-course or end of course survey  -LMS data | Descriptive statistics | N/A | LMS (n=5267), end-of-course survey (n=688) |
| Hudson L, Kortuem G, Wolff A, et al, 2016 | 2016 | Netherlands | Mixed-methods | -Pre-course survey  -Post-course or end of course survey  -LMS data  -Discussion post or learner comments | Descriptive statistics | Other qualitative | Pre-course survey (n=1067), post-course survey (n=139) |
| Study | Publication date | Country of the first author | Evaluation method | Data collection method(s) | Main data analysis method | Secondary analysis method (if any) | Group size where data was drawn from |
| Milligan C, Littlejohn A, 2014 | 2014 | UK | Qualitative | -Other survey  -Interview | Other qualitative | N/A | Survey and interview (n=35) |
| Rubio F, 2014 | 2014 | USA | Quantitative | -LMS data  -Quiz or assessment grades | Inferential statistics | N/A | LMS and Tests (pre-test and post-test) (n=50) |
| Konstan JA, Walker JD, Brooks DC, et al 2015 | 2015 | USA | Mixed-methods | -Pre-course survey  -Post-course or end of course survey  -Quiz or assessment grades (pre-test and post-test and 5 months post MOOC test) | Inferential statistics | N/A | LMS (n=6019), pre-course survey (n=4844), pre-class test (n= 4844), post-course knowledge test (n=314), retention test (n=97), |
| Morales M, Rizzardini RH, Gütl C, 2014 | 2014 | Guatemala | Quantitative | -Post-course or end of course survey  -LMS data | Descriptive statistics | N/A | LMS (n=1524), post course survey (n=584), drop-out survey (n=9808) |
| Tawfik AA, Reeves TD, Stich AE, et al, 2017 | 2017 | USA | Mixed-methods | -Discussion posts | Thematic analysis (Content analysis) | Social network analysis | Discussion posts (n=277 learners) |
| Study | Publication date | Country of the first author | Evaluation method | Data collection method(s) | Main data analysis method | Secondary analysis method (if any) | Group size where data was drawn from |
| Jacquet GA, Umoren RA, Hayward AS, et al, 2017 | 2017 | USA | Quantitative | -LMS data  -Quiz or assessment grades | Inferential statistics | N/A | LMS (n=5935), pretest and posttest 2016 (n=194), pretest and posttest 2017 (n=81) |
| Mee CK, Mei Sui LK, Jano Z, et al, 2016 | 2016 | Malaysia | Qualitative | -Interview | Thematic analysis (Grounded theory) | N/A | Interview (n=10) |
| Alario-Hoyos C, Estévez-Ayres I, Pérez-Sanagustín M, et al, 2017 | 2017 | Spain | Quantitative | -Other survey | Descriptive statistics | N/A | Survey (n=6335) |
| Liu M, Kang J, Cao M, et al, 2014 | 2014 | USA | Mixed-methods | -Other survey  -Interview  -LMS data | Thematic analysis (Focused coding) | N/A | Survey (n=409), Interview (n=44) |
| Shapiro HB, Lee CH, Wyman Roth NE, et al, 2017 | 2017 | USA | Mixed-methods | -Interview | Sentiment analysis | Thematic analysis | Interview (n=36), survey (n=36) |
| Milligan C, Littlejohn A, 2017 | 2017 | UK | Qualitative | -Other survey | Other qualitative (emergent coding) | N/A | Survey (n=970) |
