# Supplementary material for: Massive Open Online Course Evaluation Methods: Systematic Review
Source: J Med Internet Res. 2020 Apr 27;22(4):e13851. doi: 10.2196/13851 (PMC7215503; doi:10.2196/13851)
Supplement: Multimedia Appendix 4 [file jmir_v22i4e13851_app4.docx]

**Multimedia Appendix 4:**

Quality assessment results of the Randomized Controlled Trial [20] using the Cochrane Collaboration Risk of Bias Tool

| Domain | Author's judgement | Support for Judgement |
| --- | --- | --- |
| Selection bias: Random sequence generation | low | Quote: "a randomised allocation schedule was computer generated  by an independent person in Australia. The schedule was blocked  and stratified by year of study (second-year student versus third year  student). " Additionally, used intention-to-treat analysis. |
| Selection bias Allocation concealment | low | Quote: "Each participant was individually emailed to reveal group allocation and to provide instructions about what they were expected to do." |
| Performance bias Blinding (participants and personnel) | High | Open label. |
| Detection bias Blinding (outcome assessment) | High | Open label. However, researchers tried to blind participants by asking them not to discuss their allocations with others. Participants were also not informed about the purpose of the trial and were not informed whether their group was "experimental" or "control" group. Additionally, blinding was assessed by asking participants the following questions: "the participants  were asked at the end of the trial the following question: ‘The  hypothesis of this trial was that the experimental group would do  better than the control group. Which group do you think you  belonged to?’" |
| Attrition bias Incomplete outcome data | low | All randomised participants outcome data was included. There were no dropouts or incomplete data. |
| Reporting bias Selective reporting | low | All pre-specified outcomes were reported |
| Other bias Other sources of bias | low | Bias of using only one method to test the learning and motivation. Also using self-reported data only. |
