# Supplementary material for: Massive Open Online Course Evaluation Methods: Systematic Review
Source: J Med Internet Res. 2020 Apr 27;22(4):e13851. doi: 10.2196/13851 (PMC7215503; doi:10.2196/13851)
Supplement: Multimedia Appendix 5 [file jmir_v22i4e13851_app5.docx]

**Multimedia Appendix 5:**

Quality assessment results of cross-sectional studies using the NIH - National Heart, Lung and Blood Institute quality assessment tool

| Study | Rating |
| --- | --- |
| Li Q, Wan F. 2016 [40] | Poor |
| Lei C-U, Hou X, Kwok TTO, et al. 2015 [49] | Poor |
| Singh AB, Mørch AI. 2018 [34] | Poor |
| Liang D, Jia J, Wu X, et al. 2014 [39] | Poor |
| Chen W, Jia J, Miao J, et al, 2015 [31] | Poor |
| Lin J, Cantoni L. 2017 [53] | Poor |
| Alemán de la Garza LY, Sancho-Vinuesa T, Gómez Zermeño MG. 2015 [47] | Poor |
| Khalil M, Ebner M. 2018 [27] | Fair |
| Draffan EA, Leon M, James A, et al. 2018 [46] | Poor |
| Kaveri A, Gunasekar S, Gupta D, et al. 2016 [22] | Poor |
| Alturkistani A, Car J, Majeed A, et al. 2018 [41] | Poor |
| Lesjak B, Florjan V. 2014 [25] | Poor |
| Cross, Simon 2013 [42] | Poor |
| Liu M, Kang J, McKelroy E. 2015 [37] | Poor |
| Brunton J, Brown M, Costello E, et al, 2017 [48] | Poor |
| Mackness J, Waite M, Roberts G, et al, 2013 [35] | Poor |
| Colvin KF, Champaign J, Liu A, et al, 2014 [32] | Fair |
| MacKay JRD, Langford F, Waran N, 2016 [30] | Poor |
| Stephens M, Jones KML, 2014 [54] | Poor |
| Warriem JM, Murthy S, Iyer S, 2016 [43] | Poor |
| Hudson L, Kortuem G, Wolff A, et al, 2016 [44] | Poor |
| Milligan C, Littlejohn A, 2014 [55] | Poor |
| Rubio F, 2014 [21] | Poor |
| Konstan JA, Walker JD, Brooks DC, et al 2015 [33] | Fair |
| Morales M, Rizzardini RH, Gütl C, 2014 [26] | Poor |
| Tawfik AA, Reeves TD, Stich AE, et al, 2017 [50] | Fair |
| Jacquet GA, Umoren RA, Hayward AS, et al, 2018 [64] | Poor |
| Mee CK, Mei Sui LK, Jano Z, et al, 2016 [51] | Poor |
| Alario-Hoyos C, Estévez-Ayres I, Pérez-Sanagustín M, et al, 2017 [28] | Poor |
| Liu M, Kang J, Cao M, et al, 2014 [38] | Poor |
| Shapiro HB, Lee CH, Wyman Roth NE, et al, 2017 [36] | Fair |
| Milligan C, Littlejohn A, 2017 [24] | Poor |
