# Supplementary material for: Massive Open Online Course Evaluation Methods: Systematic Review
Source: J Med Internet Res. 2020 Apr 27;22(4):e13851. doi: 10.2196/13851 (PMC7215503; doi:10.2196/13851)
Supplement: Multimedia Appendix 6 [file jmir_v22i4e13851_app6.docx]

**Multimedia Appendix 6:**

Quality assessment results for the quasi experimental study using the Cochrane Collaboration Risk of Bias Tool for Before-After (Pre-Post) Studies With No Control Group

| Study | Rubio F, 2015 [21] |
| --- | --- |
| 1. Was the study question or objective clearly stated? | Yes |
| 2. Were eligibility/selection criteria for the study population prespecified and clearly described? | Yes |
| 3. Were the participants in the study representative of those who would be eligible for the test/service/intervention in the general or clinical population of interest? | Yes |
| 4. Were all eligible participants that met the prespecified entry criteria enrolled? | CD |
| 5. Was the sample size sufficiently large to provide confidence in the findings? | No |
| 6. Was the test/service/intervention clearly described and delivered consistently across the study population? | Yes |
| 7. Were the outcome measures prespecified, clearly defined, valid, reliable, and assessed consistently across all study participants? | Yes |
| 8. Were the people assessing the outcomes blinded to the participants' exposures/interventions? | Yes |
| 9. Was the loss to follow-up after baseline 20% or less? Were those lost to follow-up accounted for in the analysis? | Yes |
| 10. Did the statistical methods examine changes in outcome measures from before to after the intervention? Were statistical tests done that provided p values for the pre-to-post changes? | Yes |
| 11. Were outcome measures of interest taken multiple times before the intervention and multiple times after the intervention (i.e., did they use an interrupted time-series design)? | No |
| 12. If the intervention was conducted at a group level (e.g., a whole hospital, a community, etc.) did the statistical analysis take into account the use of individual-level data to determine effects at the group level? | Yes |
| Quality rating | Good |
